# Factors affecting IgG4-mediated complement activation

**DOI:** 10.3389/fimmu.2023.1087532

**Published:** 2023-01-26

**Authors:** Nienke Oskam, Timon Damelang, Marij Streutker, Pleuni Ooijevaar-de Heer, Jan Nouta, Carolien Koeleman, Julie Van Coillie, Manfred Wuhrer, Gestur Vidarsson, Theo Rispens

**Affiliations:** ^1^ Sanquin Research and Landsteiner Laboratory, Department of Immunopathology, Academic Medical Center, Amsterdam, Netherlands; ^2^ Department of Immunohematology Experimental, Sanquin Research, Amsterdam, Netherlands; ^3^ Department of Biomolecular Mass Spectrometry and Proteomics, Utrecht Institute for Pharmaceutical Sciences and Bijvoet Center for Biomolecular Research, Utrecht University, Utrecht, Netherlands; ^4^ Center for Proteomics and Metabolomics, Leiden University Medical Center, Leiden, Netherlands

**Keywords:** antibodies, glycoengineering, fab arm exchange, IgG4-related disease, primary membranous nephropathy, complement activation

## Abstract

Of the four human immunoglobulin G (IgG) subclasses, IgG4 is considered the least inflammatory, in part because it poorly activates the complement system. Regardless, in IgG4 related disease (IgG4-RD) and in autoimmune disorders with high levels of IgG4 autoantibodies, the presence of these antibodies has been linked to consumption and deposition of complement components. This apparent paradox suggests that conditions may exist, potentially reminiscent of *in vivo* deposits, that allow for complement activation by IgG4. Furthermore, it is currently unclear how variable glycosylation and Fab arm exchange may influence the ability of IgG4 to activate complement. Here, we used well-defined, glyco-engineered monoclonal preparations of IgG4 and determined their ability to activate complement in a controlled system. We show that IgG4 can activate complement only at high antigen and antibody concentrations, *via* the classical pathway. Moreover, elevated or reduced Fc galactosylation enhanced or diminished complement activation, respectively, with no apparent contribution from the lectin pathway. Fab glycans slightly reduced complement activation. Lastly, we show that bispecific, monovalent IgG4 resulting from Fab arm exchange is a less potent activator of complement than monospecific IgG4. Taken together, these results imply that involvement of IgG4-mediated complement activation in pathology is possible but unlikely.

## 1 Introduction

**Figure 1:**
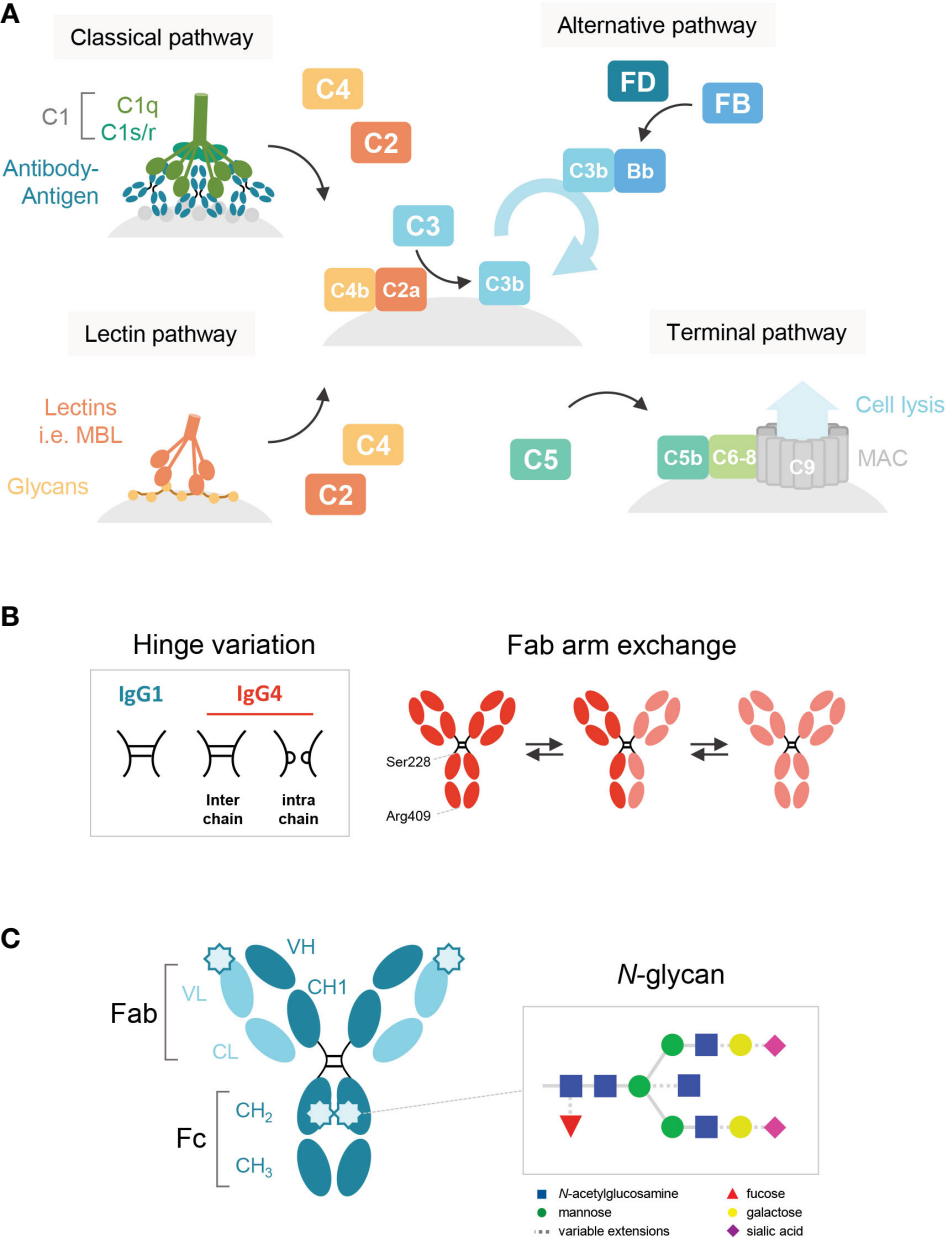
Schematic overview of the human IgG antibody structure and antibody-mediated complement activation. **(A)** Generalized overview of the complement system, which is activated through three pathways: the classical, lectin and alternative pathway. The classical pathway is activated by binding of C1q to antigen-bound IgM and IgG, which then forms the C1 complex with its proteases C1r/s. The lectin pathway is activated by the binding of lectins, such as mannose-binding lectin (MBL), to carbohydrates on cell surfaces. C3b deposition induced by these pathways or spontaneous hydrolysis of C3 can activate the alternative pathway, mediated by Factor B and D, which results in an amplification loop of C3b deposition. C3b can opsonize the target cell for phagocytosis and can lead to the assembly of the membrane attack complex with factors C5b to C9 through the terminal pathway and subsequent cell lysis. **(B)** A structural representation of the hinge variation and half molecule exchange of IgG4 *via* switch of inter-chain to intra-chain disulfide bonds at amino acid positions 228 and 409. **(C)** General structure of an IgG monomer and schematic representation of *N*-linked glycan composition of human IgG Abs. The glycans are attached to asparagine (N) at position 297 in the C_H_2 domain and have a biantennary heptasaccharide core (solid line) and variable extensions (dash line), such as fucose, galactose and/or sialic acid. Novel glycosylation sites can be introduced within the Fab region of matured antibodies.

Immunoglobulin G 4 (IgG4) is the least prevalent of the four human IgG subclasses and unique in that it seems to function mainly as an anti-inflammatory, blocking antibody. In contrast to IgG1 and IgG3, the most potent subclasses in activating complement *via* the classical pathway and inducing complement-dependent cytotoxicity (CDC; **Figure 1A**), IgG4 is generally considered to be either inactive (1–4) or a poor activator (5–7). IgG4 may interfere with multimerization and complement component 1q (C1q) binding of the other subclasses, and thereby effectively block complement activation (6, 8). It has been hypothesized that the impaired ability of IgG4 to activate complement is due to a conformational change of the FG loop in the C_H_2 domain that may interfere with C1q binding (3, 4, 9).

Despite the consensus on the impaired ability of IgG4 to activate complement, IgG4 levels have been associated with pathologies in IgG4-related disease (IgG4-RD) and in several autoimmune disorders, such as pemphigus, autoimmune pancreatitis, and primary membranous nephropathy (pMN) (10–12). Often, deposits of both IgG4 antibodies and complement components can be detected in biopsies of affected tissues and correlate to serum level, IgG4 titers and hypocomplementemia (10–12). For example, most patients suffering from pMN (~70%) develop IgG4 autoantibodies towards PLA_2_R1, a secretory phospholipase A2 receptor, expressed on cells in the glomerular basement membrane of the kidney (13). Although reports vary, deposited C4d has been found in 84-100% of biopsies in pMN (10), which implicates complement activation in the pathogenesis, but its significance and relation to the presence of IgG4 autoantibodies remain debated.

This raises several questions that warrant further investigation. Even though IgG4 has been considered a poor activator of complement at best, there could be certain conditions, potentially reminiscent of *in vivo* deposits, in which complement activation can be observed. Furthermore, IgG4 undergoes several post-translational modifications, such as glycosylation and Fab arm exchange, that have been implicated in complement activation, but not studied in detail for IgG4 despite the importance of these modifications to its function.

Fab arm exchange is a unique aspect of IgG4 and refers to its ability to exchange half-molecules of one heavy chain-light chain pair with another IgG4 molecule (**Figure 1B**). Fab arm exchange leads to the formation of a bispecific antibody which remains functionally monovalent as the antibody is unable to crosslink antigens (14–17). Therefore, IgG4 is effectively unable to form large immune complexes. It has been shown for IgG1 that functional monovalence may enhance complement activation (18), but despite the importance of Fab arm exchange in IgG4 biology, its importance for complement activation has never been studied.

IgG antibodies contain a conserved *N*-linked glycan site, asparagine (N) at the position 297, in both C_H_2 domains of the Fc region (**Figure 1C**). The core structure of the IgG *N*-linked glycans comprises N-acetylglucosamine (GlcNAc) and mannose residues with possible extensions of galactose, sialic acid, core fucose, and bisecting GlcNAc (1, 19). Several studies have shown that the exact composition of the Fc glycan can either enhance or reduce the potency of IgG1 to activate complement (20–22). Galactosylation in particular has been shown to enhance IgG1 hexamerization and therefore its ability to activate complement (23). Furthermore, upon somatic hypermutation, glycosylation sites may arise within the variable regions as well (24) and levels of Fab glycosylation are elevated for IgG4 relative to other IgG subclasses (25). Although the effect of Fab glycosylation on complement activation is not entirely clear, the presence of these glycans has been shown to influence effector molecule binding and stability of the antibody (26).

Next to the classical and alternative pathways, the complement system can also be activated *via* the lectin pathway, which is initiated by recognition of sugars often found on pathogens by either mannose-binding lectin (MBL) or other ficolins and collectins (27, 28). Although these lectins usually bind to foreign sugars, they may also bind to aberrant glycans on human proteins. It has been suggested that IgG antibodies lacking the terminal galactose residue on their Fc-glycan could activate complement *via* the lectin pathway, as MBL can bind to terminal fucose, mannose and *N*-acetylglucosamine residues, but not galactose (29). Interestingly, in both IgG4-RD and pMN, agalactosylated IgG4 (auto)antibodies have been reported (30–33). For pMN specifically, it was recently shown that MBL could bind agalactosylated IgG4 autoantibodies towards PLA_2_R1 which may activate the lectin pathway (33).

In this study, we not only present conditions that allow IgG4 to activate complement, but also show for the first time the effects of differential Fc glycosylation and Fab arm exchange on the ability of IgG4 to activate complement. We demonstrate that IgG4 can only activate the classical pathway in conditions with high antigen densities and antibody concentrations, which is further enhanced by Fc galactosylation, and that agalactosylated IgG4 does not activate the lectin pathway like suggested before. Furthermore, as Fab arm exchange reduces complement activation by IgG4, it seems that this feature would limit the capacity of IgG4 to complement activation *in vivo*.

## 2 Materials and methods

### 2.1 Production of recombinant antibodies

Recombinant IgG antibodies specific for biotin [bt; (34, 35)] and trinitrophenyl [TNP; (36)] were produced using FreeStyle™ HEK293-F cells (ThermoFisher), which were cultured in FreeStyle™ 293 Expression Medium (ThermoFisher). Using Polyethylenimine-MAX, pcDNA3.1 expression vectors encoding human κ, IgG1 and IgG4 were co-transfected under serum-free conditions into HEK293-F cells according to the manufacturer’s protocol (Invitrogen). In the case of the anti-TNP antibodies, an supplemental vector was used with a mutation of D86N in the light chain to introduce an additional glycosylation site (37). 0.9% w/v Peptone Primatone^®^ RL (Sigma-Aldrich) was added at least 4 h post transfection. The galactose-glycovariants were produced as described previously (19). To produce low galactose-glycovariants, 0.5 mM of the galactose analogue 2-deoxy-2-fluoro-d-galactose (2FG) (Carbosynth) was added 4 h post transfection. To produce high galactose-glycovariants, 5 mM D-galactose (Sigma-Aldrich) was added to the cell culture 1 h before transfection, and cells were co-transfected with 1% DNA (of total DNA transfected) encoding the β-1,4-galactosyltransferase 1 (B4GALT1).

High mannose-glycovariants were produced using HEK293-6E cells with a MGAT1 knock-out (KO), which lack N-acetylglucosaminyl transferase I (GlcNAc-T I) (38, 39). These cells were kindly provided by Prof. H. Clausen from the Copenhagen Center for Glycomics, University of Copenhagen, Denmark. The MGAT1-KO cells were cultured in FreeStyle™ F17 Expression Medium (ThermoFisher) supplemented with 4 mM L-glutamine (Sigma-Aldrich) and 0.1% w/v Kolliphor^®^ P 188 (Sigma-Aldrich), at 5% CO_2_, 37°C while shaking at 125 rpm.

Culture supernatant was harvested five or six days after transfection and filtered using a 0.2 nm Puradisc™ syringe filter (Whatman, GE Healthcare). Antibodies were purified on a HiTrap^®^ protein G HP 1 mL column (GE Healthcare) and eluted with 0.1 M glycine pH 2.5 (Merck). The eluate was then dialyzed against 5 mM NaAc (pH 4.5). Antibody concentrations were determined by measuring A280 on a NanoDrop One spectrophotometer (ThermoFisher) and samples were stored at -20°C.

### 2.2 Fab arm exchange

To be able to produce bispecific IgG1 antibodies, a mutation of K409R was introduced into the heavy chain of the IgG1 anti-bt clone, which allows for Fab arm exchange of IgG1 in a manner similar to IgG4. IgG1 K409R anti-bt was reacted with an in-house generated IgG1 K409R variant of adalimumab, and IgG4 anti-bt or anti-TNP with IgG4 natalizumab (TYSABRI^®^), both in a ratio of 1:4 (anti-bt or anti-TNP to irrelevant clone). Fab arm exchange was induced by mild reduction with 10 and 0.5 mM L-glutathione (GSH; Sigma-Aldrich) for IgG1 and IgG4 respectively, in presence of 0.1% w/v Kolliphor^®^ P407 (BASF). Samples were incubated at 37°C for over 20 h, after which the reaction was stopped with 12 mM and 0.6 mM 2-iodoacetamide (IAM; Merck) for IgG1 and IgG4 respectively. IgG1 and IgG4 bispecific antibodies were made fresh for each experiment due to aggregation of the antibodies during storage. Bispecificity was confirmed for all clones.

### 2.3 Analysis of glycosylation of antibodies

The purified glycoengineered IgG1 and IgG4 were dried by vacuum centrifugation and subjected to tryptic cleavage followed by LC-MS as described previously (40, 41). The raw LC-MS spectra were converted to mzXML files and LaCyTools, an in-house developed software, was used for the alignment and targeted extraction of raw data. The analyte list for targeted extraction of the 1+, 2+, 3+ and 4+ charge states was based on manual annotation as well as on literature reports (40). The inclusion of an analyte for the final data analysis was based on quality criteria including signal-to-noise (higher than 9), isotopic pattern quality (less than 25% deviation from the theoretical isotopic pattern), and mass error (within a ±20 parts per million range) leading to a final analyte list (**Table S1** and **S2**). Relative intensity of each glycopeptide in the final analyte list was calculated by normalization the glycopetide value to the sum of the total area of all glycopeptides included. Normalized intensities were used to calculate fucosylation, bisection, galactosylation and sialylation levels (**Table S3**).

To study the influence of glycosylation on the molecular weight of IgG1 and IgG4, a reduced SDS-PAGE was performed on a NuPAGE 4-12% Bis-Tris gel according to manufacturer’s protocol (Invitrogen). The gel was stained with InstantBlue^®^ Coomassie Protein Stain (Abcam) for 1 h and the bands were visualized using a ChemiDoc™ Universal Hood III (Bio-Rad).

### 2.4 C3b deposition ELISA

To assess complement activation by IgG1 and IgG4 variants, C3b deposition was measured in ELISA as described previously (42). In short, human serum albumin (HSA; Albuman, Sanquin) was either biotinylated with different concentrations (30 to 240 µM) of biotin (EZ-Link™ Sulfo-NHS-LC-Biotin No-Weigh; ThermoFisher) in PBS or TNPlated with 1 mM of 2,4,6-trinitrobenzenesulfonic acid (TNBS, Sigma-Aldrich) in 0.2 M Na_2_HPO_4_ (Merck, F173578238). Nunc MaxiSorp™ flat-bottom plates (ThermoFisher) were coated with 5 µg/mL of biotinylated HSA (HSA-bt) or 10 µg/mL of TNPlated HSA (HSA-TNP) in PBS and incubated at 4°C overnight. IgG antibodies were serially diluted in PBS supplemented with 0.1% v/v Tween^®^-20 and 0.2% w/v gelatin (Merck) and incubated shaking at room temperature for at least 1 h. After washing, C3b deposition was allowed for 1 hour at RT by addition of 2.5% normal human serum (NHS; pool of minimum healthy donors) in veronal buffer (3 mM barbital (Fagron), 1.8 mM sodium-barbital (Fagron), 0.146 M sodium chloride (Merck)) supplemented with 1 mM calcium chloride (CaCl_2_, Merck), 0.5 mM magnesium chloride (MgCl_2_, Merck) and 0.2% Tween-20 (VB+/+). For inhibition conditions, VB++ was supplemented with additional anti-C1q-85 (6.3 µg/mL; Sanquin (43, 44)), anti-MBL1 (0.2 µg/mL; Sanquin (45, 46)) and/or anti-complement factor D (a-FD; 0.13 µg/mL; Lampalizumab; Genentech (47, 48);) or inhouse anti- complement factor B-1 (a-FB; 24,3 µg/mL; Sanquin) in order to inhibit C1q, MBL and FD/FB respectively. All inhibitors were shown to be effective blockers of their respective route of activation (49).

Alternatively, instead of the addition of serum dilution, IgG binding to the coat was measured using mouse-anti-IgG-horseradish peroxidase (0,33 µg/mL; MH-16-1-HRP; Sanquin). Deposited C3b was detected with mouse anti-human C3-19-HRP (0.5-1 µg/mL; Sanquin (50)) and bound C1q was detected with mouse anti-human C1q-2-HRP (3 µg/mL; Sanquin (43)) and visualized using tetramethylbenzidine (TMB; Merck) mix. The reaction was stopped with 0.2 M sulfuric acid (Merck) and the optical density (OD) was measured at Δ450–540 nm using an ELISA plate reader (SynergyTM 2 Multi-Detection Microplate Reader, Biotek).

### 2.5 Complement-dependent cytotoxicity assay

The CDC assay to assess terminal pathway activation by the anti-bt IgG antibodies was performed as described earlier, with minor alterations (42). To biotinylate red blood cells (RBCs), one part packed cells was reacted with seven parts of a biotinylation reagent at a final concentration of 2.5 mM Sulfo-NHS-LC-Biotin (ThermoFisher) in PBS. RBCs were incubated at 37°C for at least 1 h with serial dilutions of anti-bt IgG antibodies, 10% of NHS pool and inhibitors (indicated for each respective experiment) in veronal buffer supplemented with 0.05% w/v gelatin, 1 mM CaCl_2_ and 0.5 mM MgCl_2_. Conditions with complement inhibitors were included as described above, but with four times higher concentrations. A positive control (100% lysis) was generated by adding 0.5% w/v saponin (Merck/Millipore) to RBCs and used to normalize the data. After incubation, 40 µL of buffer was added, plates were centrifuged at 1800 rpm for 2 min and 100 µL supernatant was transferred to MaxiSorp™ plates. The OD was measured as ΔOD(412 nm) – OD(690 nm). The percentage of cell lysis for each sample was calculated as follows: % Lysis = OD_sample_/mean OD_100%_ * 100.

### 2.6 Statistical analysis

Kruskal-Wallis test with Dunn’s multiple comparisons test was performed for statistical comparison of the IgG4 glycovariants.

## 3 Results

### 3.1 IgG4 activates complement at high densities and antibody concentrations

**Figure 2:**
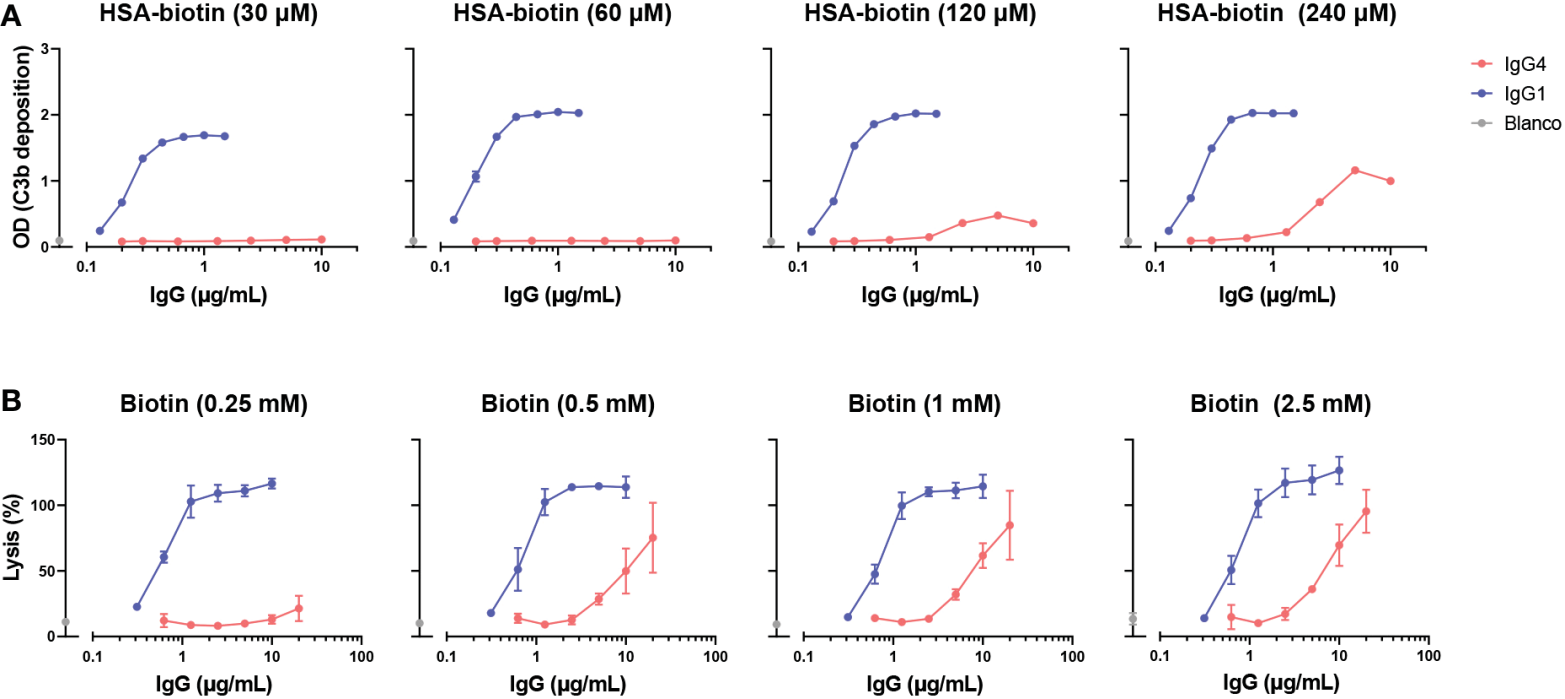
IgG4 activates complement at high antigen densities and antibody concentrations. **(A)** C3b deposition induced by anti-biotin IgG1 and IgG4 antibodies on biotinylated human serum albumin (HSA; 30, 60, 120 and 240 μM biotin; corresponding to approximately 2, 4, 8 and 50 biotins per HSA molecule respectively), in the presence of 2.5% human serum. All antibodies were tested in a two-fold serial dilution starting from 10 µg/mL for IgG4 and 2 µg/mL for IgG1 antibodies in four independent assays. **(B)** The capacity of biotin-specific IgG1 and IgG4 antibodies to induce complement-mediated lysis (as percentage) of biotinylated human red blood cells at various antigen densities (0.25, 0.5, 1, and 2.5 mM biotin) in the presence of 10% human serum. All antibodies were tested in a two-fold serial dilution starting from 20 µg/mL for IgG4 and 10 µg/mL for IgG1 antibodies in triplicates. The data was normalized to a 100% lysis control (RBCs with saponin).

To study complement activation by IgG4, C3b deposition and CDC were determined in a recombinant antibody system with anti-hapten antigens which allowed for control of antigen density. Using recombinant anti-biotin antibodies with biotinylated HSA, we found that IgG1 activated complement efficiently in all conditions tested, in contrast to IgG4 which could only activate complement at high antigen densities (HSA-bt 120 and 240 µM) and high antibody concentrations (**Figure 2A**). IgG4 required a roughly 100 times higher antibody concentration to reach the same OD as IgG1. Comparable results were obtained utilizing another antigen, TNP, where IgG4 was also only able to activate complement at high antigen densities and antibody concentrations (**Supplementary Figure 1A**). In addition, two monoclonal antibodies against protein antigens (rather than anti-hapten) were tested, anti-Betv1a (Birch pollen) and anti-idiotype Fabs (against adalimumab). No complement activation with either of these antibodies was observed, probably because sufficient densities of antibody binding were not reached (**Supplementary Figure 1B, C**).

Next, the ability of IgG4 antibodies to induce complement-dependent lysis of biotinylated RBCs was determined. In line with the results for the C3b deposition, IgG4 induced lysis at high antigen densities and antibody concentrations almost to the same extent as IgG1 antibodies (**Figure 2B**). However, IgG1 induced cell lysis at all conditions tested at much lower antibody concentrations. Taken together, this shows that IgG4 can activate complement to some extent, but only in conditions where both sufficient antigen and antibodies present.

### 3.2 IgG4 antibodies containing low levels of galactose show reduced complement activation

It has been suggested that agalactosylated IgG4 autoantibodies can induce complement activation through the lectin pathway *via* MBL (33). To study this effect of galactosylation on complement activation by IgG4 in more detail, glycoengineered variants with relatively low and high levels of galactosylation were produced. These anti-biotin IgG1 and IgG4 antibodies were classified as ‘low galactose’ (LG; 10% galactose), ‘normal galactose’ (NG; 26-40%) and ‘high galactose’ (HG; 75-80%) based on their galactosylation levels determined by mass spectrometry (**Supplementary Figure 2A**). The antibodies were also analyzed by gel electrophoresis under reducing conditions and subtle shifts in size can be observed among the heavy chains due to varying levels of galactosylation (**Supplementary Figure 2B**). As a glycan control, cells deficient for N-acetylglucosaminyl transferase 1 were used to produce ‘high mannose’ (HM) variants (38, 39). An IgG1 antibody variant (referred to as IgG1 PG-LALA) with a combination of mutations that eliminate Fc-mediated effector functions, including complement was included as a “complement-dead” control (51).

**Figure 3:**
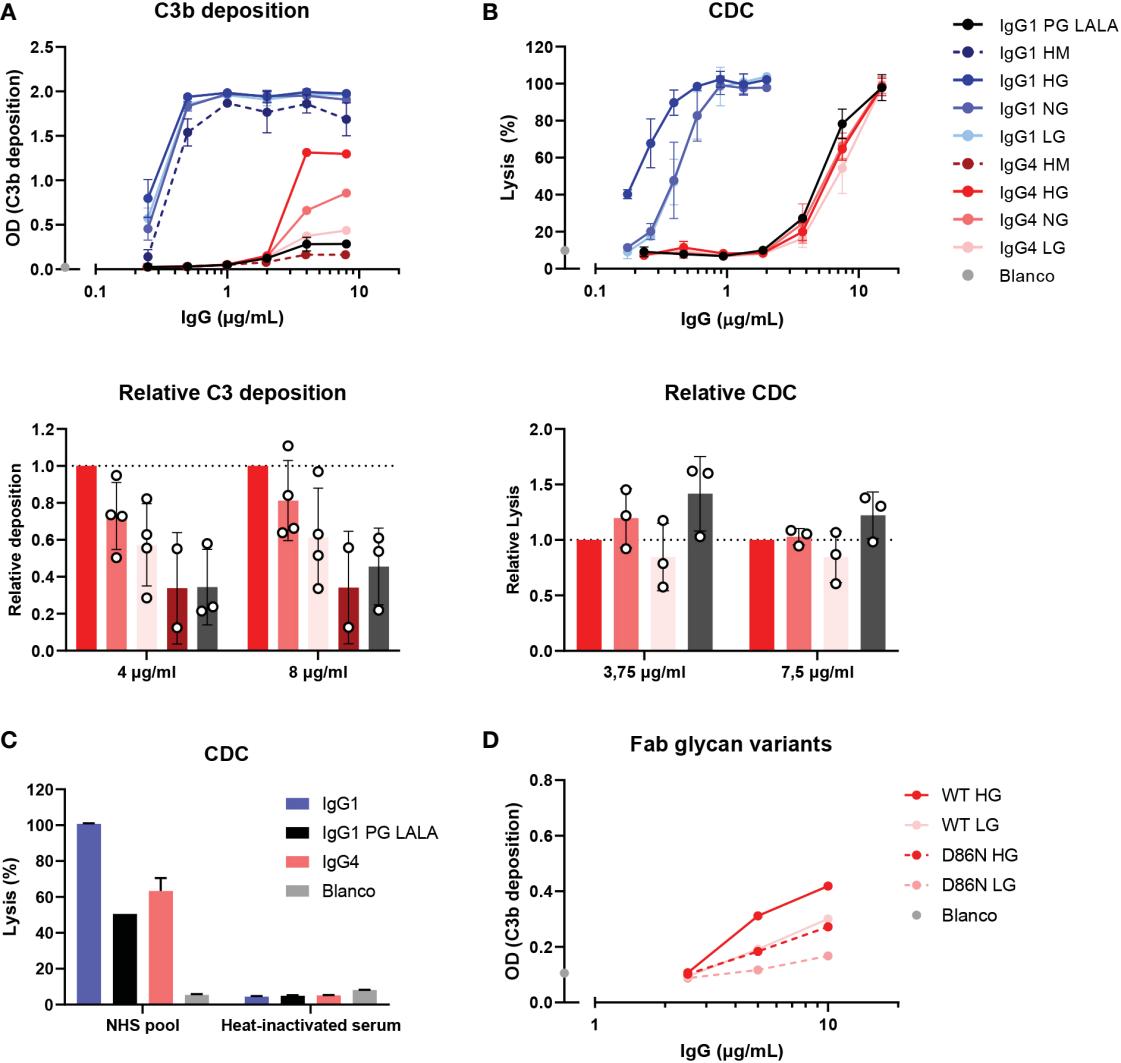
IgG4 glycovariants can induce C3b deposition to different extent. **(A)** C3b deposition induced by anti-biotin IgG1 and IgG4 glycovariants (high mannose (HM), high galactose (HG), normal galactose (NG) and low galactose (LG)), and IgG1 PG-LALA in the presence of biotinylated human serum albumin (240 μM biotin) and 2.5% human serum. Relative C3b deposition of the IgG4 glycovariants at concentrations 4 µg/mL and 8 µg/mL was normalized to IgG4 HG, which was set to 1 (dashed line), is shown as bar graphs. **(B)** Percentage of complement-mediated lysis of biotinylated human red blood cells (2.5 mM biotin) by the anti-biotin IgG1 and IgG4 glycovariants. The percentage of lysis was calculated based on the 100% lysis control (red blood cells with saponin). The percentage of complement-mediated lysis of the IgG4 glycovariants at concentrations 3.75 µg/mL and 7 µg/mL normalized to IgG4 HG is shown as bar graphs. Kruskal-Wallis test with Dunn’s multiple comparisons test of the IgG4 glycovariants was performed. **(C)** To test unspecific complement-dependent cytotoxicity (CDC), anti-biotin IgG1, IgG4 and IgG1 PG-LALA (eliminated Fc-mediated effector functions) were tested in the presence of biotinylated RBCs and 10% normal or heat-inactivated human serum. **(D)** C3b deposition induced by anti-trinitrophenyl (TNP) IgG4 glycovariants variants (HG and LG), which were produced as wild type IgG4, and a variant with D86N substitution in the light chain to introduce an additional glycosylation site in the Fab region. All experiments were performed at least three times, shown are representative figures for the C3b deposition and the mean ± standard deviation for the lysis experiments.

The capacity of these glycovariants to activate complement was determined in C3b deposition ELISAs and CDC assays at high antigen densities (biotinylated HSA with 240 μM biotin and RBCs biotinylated at 2.5 mM respectively). At the level of C3b deposition, differences between the IgG4 glycovariants were observed, with the HG variant steadily outperforming the NG and LG variants (**Figure 3A**). The differences across glycoengineered IgG1 antibodies were less pronounced, but nevertheless consistent with previous results (23), as complement activation reached saturation at even low antibody concentrations. Both IgG1 and IgG4 HM variants induced the lowest levels of C3b deposition of all variants within each subclass. IgG1 PG-LALA was not able to mediate any detectable C3b deposition when tested in similar conditions as IgG4. No deposition was found with heat-inactivated serum and the binding of all glycovariants was found to be identical (**Supplementary Figure 3A, C**). In contrast, the differences in IgG4 galactosylation did not result in similarly clear differences at the level of cell lysis (**Figure 3B**). Furthermore, IgG1 PG-LALA, which is incapable of inducing complement activation, showed similar levels of cell lysis as the IgG4 variants. For both the IgG4 antibodies as well as the IgG1 PG-LALA variant, this effect appears to be predominantly complement-mediated, as heat-inactivated serum does not cause any cell lysis (**Figure 3C** and **Supplementary Figure 3C**). Red blood cells are sensitive to lysis as the formation of a single complement membrane attack complex can already cause cell lysis, especially at very high antibody concentrations and even when the antibody is considered ‘complement-dead’. Taken together, no enhanced complement activation for hypogalactosylated IgG4 was observed, on the contrary, high levels of Fc-galactosylation resulted in increased complement activation by IgG4, which is in line with observations using differently galactosylated IgG1 (20, 21, 23, 52).

### 3.3 The presence of Fab glycans interferes with complement activation by IgG4

We next investigate the possible role of the Fab glycans on complement activation as they are relatively abundant on IgG4 (25). Therefore, WT IgG4 antibodies were produced against TNP (containing only an Fc glycan), and variants with a mutation in the variable region (D86N) of the light chain that introduces an additional glycosylation site within the Fab domain. These antibodies were generated as ‘low’ (10%) galactose and ‘high’ (70%) galactose variants and had Fc glycan composition levels like the anti-biotin variant as described above (**Supplementary Figure 1C, D**). Again, both HG variants induced more C3b deposition than the LG variants (**Figure 3D**), suggesting this is Fc-glycan mediated. The introduction of the additional glycan in the Fab interfered with the ability of the antibody to activate complement, as both antibodies with the additional Fab glycan performed worse than the WT variants. Antigen binding of all variants appears to be similar, indicating that this is not the cause of the decreased ability to activate complement. (**Supplementary Figure 3A**).

### 3.4 IgG4 antibodies activate complement *via* the classical pathway

**Figure 4:**
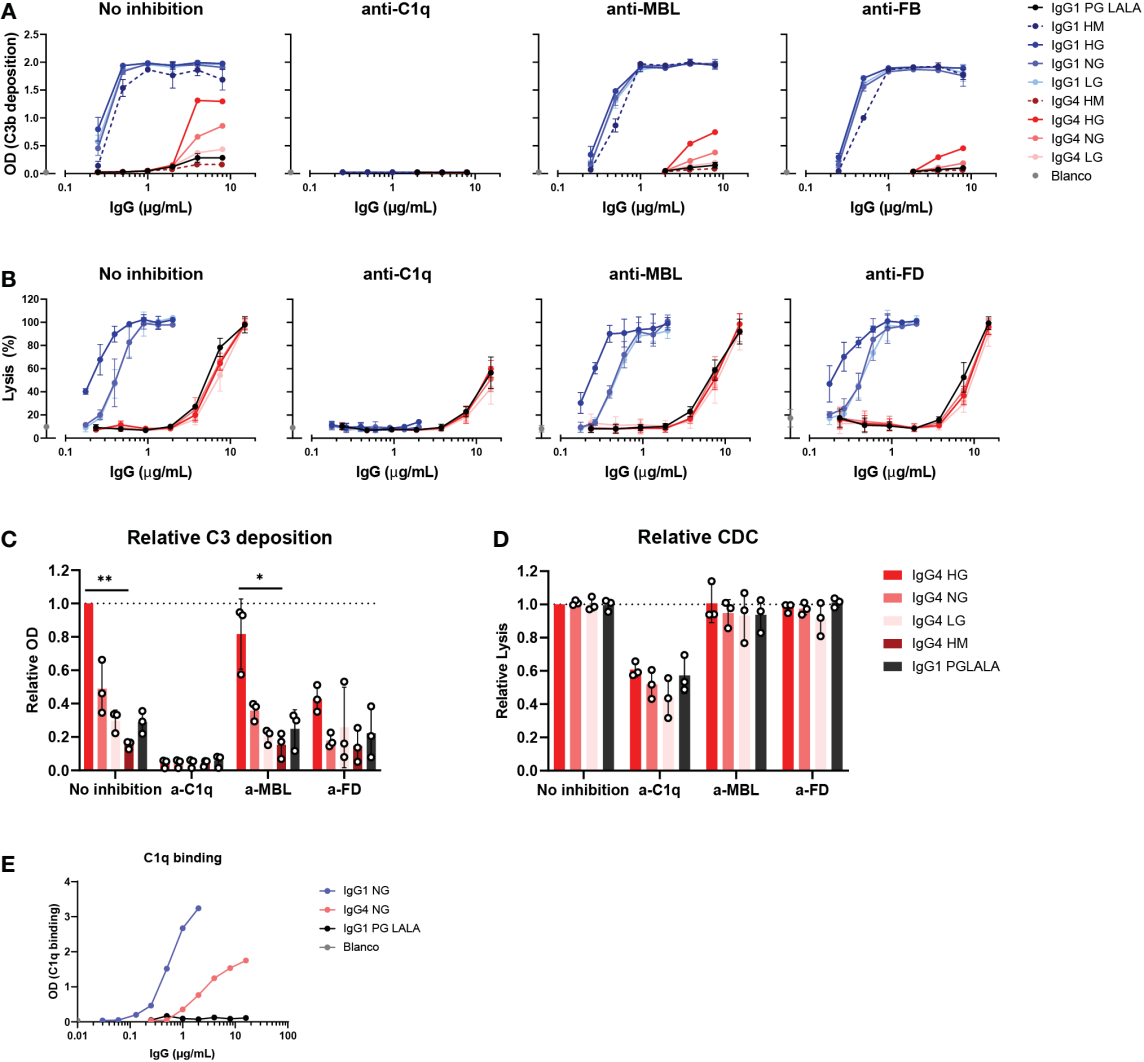
IgG4 activates complement *via* the classical pathway. **(A)** C3b deposition by anti-biotin IgG1, IgG4 and IgG1 PG-LALA (eliminated Fc-mediated effector functions) in presence or absence of either C1q, mannose-binding lectin (MBL), or complement factor B (FB) inhibitors, biotinylated human serum albumin (HSA; 240 μM biotin) and 2.5% human serum. Antibodies with different glycan profiles were tested: high mannose (HM), high galactose (HG), normal galactose (NH) and low galactose (LG). The molar ratios of target to inhibitor are 1:4.5 for C1q, 1:8.5 for MBL, and 1:3.5 for FB. **(B)** The percentage of complement-mediated lysis of biotinylated human red blood cells (2.5 mM biotin) by IgG1, IgG4 and IgG1 PG-LALA in presence or absence of C1q, MBL, or factor D (FD) inhibitors and 10% human serum. The molar ratios for target to inhibitor were 1:3 for C1q, 1:3 for MBL, and 1:2 for FD. The percentage of lysis was calculated based on the 100% lysis control (red blood cells with saponin). **(C)** The relative C3b deposition and **(D)** percentage of complement-mediated lysis of biotinylated human red blood cells induced by IgG4 glycovariants are shown as bar graphs with an antibody concentration of either **(C)** 8 µg/mL, or **(D)** 15 µg/mL. **(E)** To test binding of purified C1q (600 μg/mL) to the mAbs, IgG1, IgG4 and IgG1 PG-LALA were tested in the presence of biotinylated human serum albumin (HSA; 240 μM biotin). Kruskal-Wallis test with Dunn’s multiple comparisons test was performed for statistical comparison of the IgG4 glycovariants per inhibition condition: *p < 0.05; **p < 0.01. All antibodies were tested in a two-fold serial dilution starting from 8 µg/mL for C3b deposition and 15 µg/mL for CDC in at least three independent experiments.

Next, we investigated which pathway of the complement system was activated by these IgG4 glycovariants. C3b deposition and RBC lysis were performed in the presence of inhibitors of C1q, MBL, and FD, to determine the contribution of the classical pathway, lectin pathway and alternative pathway, respectively. Despite the suggestion that IgG4 might activate complement *via* MBL (33), we found that C3b deposition by all IgG1 and IgG4 glycovariants was fully inhibited when blocking C1q, and not, or only slightly, in case of blocking MBL or FD (**Figure 4A, C**). This indicated that the main mode of activation of IgG4 is *via* the classical pathway and moreover, that this activation is irrespective of glycosylation as all variants rely on C1q for activation. Moreover, we found that C1q could also bind to IgG4, although to a lesser extent than to IgG1 (**Figure 4E**). Inhibition of FD decreased C3b deposition slightly compared to the condition without any inhibitor, suggesting that the alternative pathway may amplify activation *via* the classical pathway. As the alternative pathway requires higher serum concentrations to be studied, we also performed the experiment at 10% serum and allowed deposition at 37°C and we found similar results (**Supplementary Figure 3D**). No clear inhibition was found when MBL was blocked. The blocking antibody was able to completely block mannan-induced C3b deposition at equal serum concentrations, showing that the antibody is effective (**Supplementary Figure 4A**). Furthermore, when the assay was repeated with serum of an MBL deficient donor, IgG4 was still able to activate complement (**Supplementary Figures 4A, B, D**) The glycovariants were not affected differently by any of the inhibitions. Similar results at the level of cell lysis were observed: C1q inhibition significantly reduced IgG4-mediated lysis (**Figures 4B, D**), whereas inhibition of either MBL or FD had no impact on the induced lysis by IgG4. Furthermore, IgG4 could still induce cell lysis with serum from an MBL deficient donor (**Supplementary Figure 4C**).

Taken together, no role for the lectin pathway in IgG4 complement activation was found, which instead appears to mainly employ the classical pathway that was slightly enhanced by galactosylation on the level of C3b deposition.

### 3.5 Complement activation by IgG4 antibodies is reduced upon fab arm exchange

**Figure 5:**
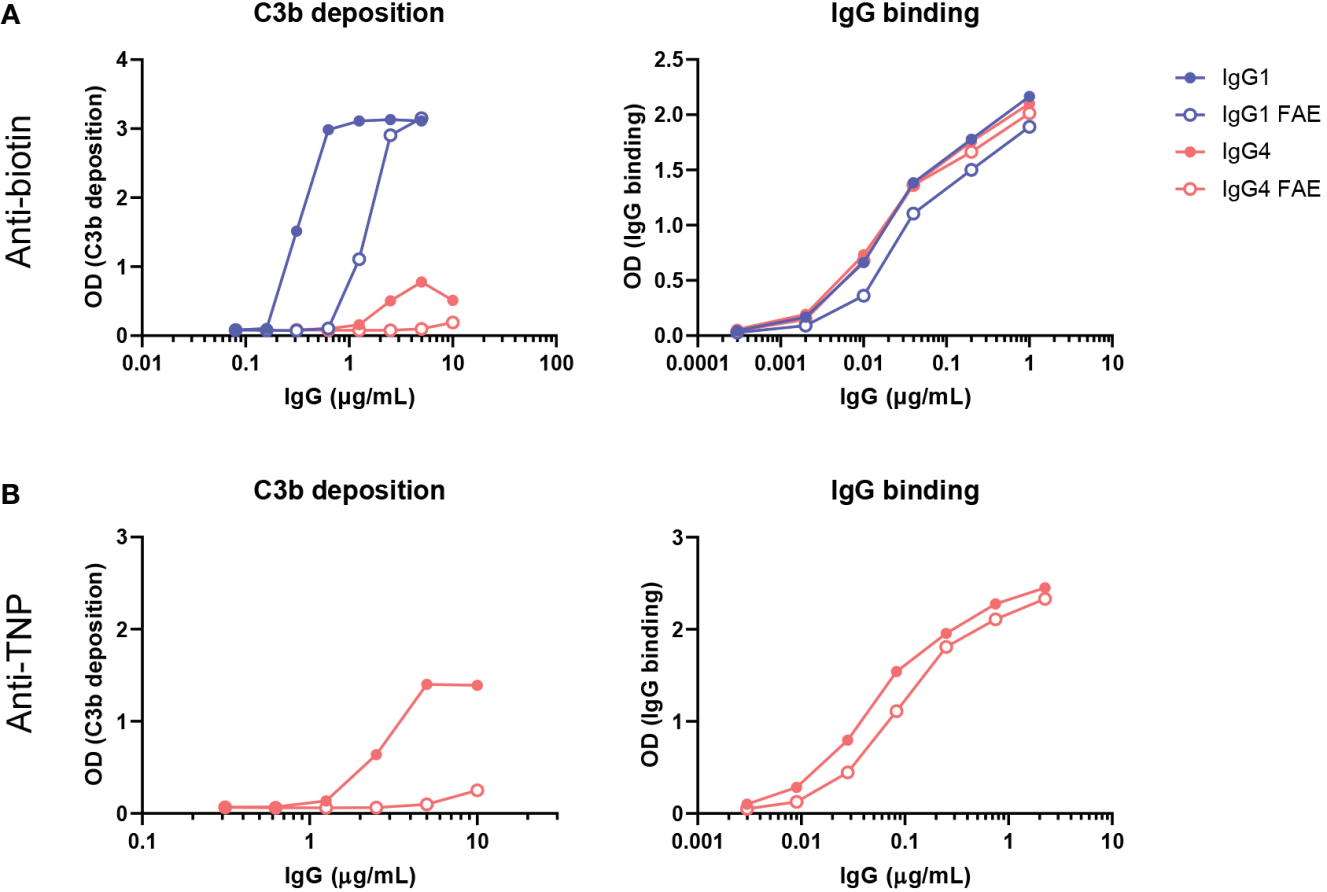
Fab arm exchange reduces antibody-mediated C3b deposition. Bispecific IgG1 and IgG4 variants were generated by half molecule exchanging anti-biotin and anti-trinitrophenyl (TNP) clones (both normal galactosylation) with IgG1 K409R adalimumab and IgG4 natalizumab respectively under mild reduction conditions. C3b deposition by **(A)** anti-biotin and **(B)** anti-TNP monospecific (full dots) and bispecific (empty dots) IgG1 and IgG4 in the presence of either TNPlated (1 mM 2,4,6-trinitrobenzenesulfonic acid) or biotinylated (240 μM biotin) human serum albumin and 2.5% human serum was tested. Binding to either biotin or TNP was measured for all variants (right panel). All experiments were performed at least three times, shown are representative figures.

In our previous assays, only bivalent-monospecific antibodies were used, but IgG4 antibodies can recombine half-molecules with different antigen specificities *in vivo*. Through this Fab arm exchange process, monovalent-bispecific IgG4 antibodies with decreased avidity for their targets are generated (15). We therefore exchanged IgG4 monoclonal anti-biotin and anti-TNP half-molecules with an irrelevant clone (natalizumab with no target in our assays) to create a bispecific antibody that would bind to biotin and TNP respectively with only one arm. In addition, a K409R mutation in the IgG1-Fc was introduced, which allowed for the IgG1 anti-biotin clone to be exchanged with an adalimumab clone (also no target in our assays) with the same mutation. For the fab arm exchange process mild reduction conditions are necessary. These antibodies were then tested for C3b deposition *via* ELISA, which was found to be reduced for both anti-biotin and anti-TNP bispecific IgG4 clones, but also for the bispecific anti-biotin IgG1 clone in comparison to their monospecific counterparts. The binding of the monospecific and bispecific IgG antibodies to either biotin (**Figure 5A**) or TNP (**Figure 5B**) was comparable for parental (mock condition) and Fab arm exchange clones. To conclude, this shows that Fab arm exchange greatly reduces the capacity of IgG4 to induce complement activation.

## 4 Discussion

IgG4 is generally considered a poor activator of complement, but at the same time has been linked to complement-induced fibrosis in IgG4-RD and IgG4 autoimmunity (10–12). This paradox prompted us to extensively investigate complement activation by IgG4, by searching for conditions that allowed for IgG4-induced complement activation and subsequently study how different features of IgG4 influenced this activation. These included glycosylation and Fab arm exchange using well-defined, glycoengineered, recombinant monoclonal antibodies against both protein and hapten antigens, which allowed systematic exploration of a wide range of experimental conditions.

Agalactosylated IgG has been shown to correlate with disease severity in several autoimmune diseases where complement contributes to pathology, including rheumatoid arthritis and systemic lupus erythematosus (53, 54). Agalactosylated IgG4 specifically has been implicated in IgG4-RD (31, 32) and pMN (30, 33). From there it was hypothesized that these agalactosylated IgG4 autoantibodies may be bound by MBL and could therefore activate complement *via* the lectin pathway (10, 12). However, PLA_2_R1-associated pMN can also occur in individuals with MBL deficiency (55) and IgG4 anti-PLA_2_R1 antibodies often co-occur with other subclasses as well (56, 57). In accordance, we found that the lectin pathway did not contribute to complement activation by IgG4 in our system. Neither the agalactosylated, nor high mannose glycovariants employed markedly different routes of activation, but also inhibition of MBL did not affect overall complement activation. Moreover, inhibition of C1q abolished complement activation, indicating that IgG4 in general, but also all tested glycovariants activate complement predominantly, if not exclusively, *via* the classical pathway. Lastly, variable domain glycosylation, known to be elevated in IgG4 (25), diminished rather than enhanced complement activation of IgG4. These findings are in contrast with recent findings showing that IgG4 anti-PLA_2_R1 antibodies from pMN patients could be bound by MBL and the suggestion that the lectin route is mainly responsible for complement activation at affected tissues (33). However, the authors also showed that upon removal of the Fc-glycan with the enzyme PGNase F, MBL was still able to bind to IgG4, which suggests that the reported binding of MBL was not dependent on the glycans on IgG4. Another recent study reported that the activation of the alternative pathway by IgG4 autoantibodies is sufficient in pMN (58), whereas the contribution of the alternative pathway was only minor in our experiments.

It has been previously described that at high concentrations of both antibody and antigen, IgG4 could induce C3 and terminal pathway activation (4, 59). We confirm that IgG4 can activate complement at high antigen densities and high antibody concentrations and show for the first time that this activation is mainly dependent on the classical pathway. This contradicts other studies which showed that IgG4 is effectively complement-dead. It is likely that these effects are missed in other studies, since IgG1 and IgG3 are much more efficient in activating complement and therefore comparable conditions with high levels of antigens and antibodies were often not included in comparative studies of IgG subclasses (21, 60, 61). The importance of the antigenic context is further illustrated by the fact that we only found complement activation by monoclonal IgG4 antibodies using haptens (biotin and TNP), but not protein antigens (Betv1a and anti-ADL fabs). This feature is likely to be the result of the high effective antigen densities accommodated by high hapten density in combination with high levels of antibodies. Having such high hapten density may possibly reflect a polyclonal antibody response targeting multiple epitopes on a protein target more closely. Whether similar conditions are met *in vivo*, for example within antibody deposits in IgG4-RD or pMN, remains to be investigated.

Upon recruiting antibodies to a surface *via* antigen binding, IgG Fc tails can cluster together to form hexamers *via* weak Fc-Fc interactions, mediated in part by Fc glycans, to interact with the hexagonal C1 complex (18, 62, 63). C1q has a low affinity to singular IgG molecules and requires multiple IgG-Fc tails to be in proximity and form oligomers to bind (64, 65). Despite similarities in the Fc-tails among the different IgG subclasses, IgG4 interacts poorly with C1q due to a variation in position 331, which has been shown to directly reduce binding of C1q (66, 67). Regardless, IgG4 is not completely unable to bind C1q. A fusion of the IgM tail piece to IgG4, which causes it to multimerize, greatly improved the ability of IgG4 to induce lysis (68). Similarly, this was also shown for the IgG hexamer-enhancing mutation E430G (18, 61). This suggests that although binding of C1q to IgG4 is reduced as compared to the other isotypes, this can partly be overcome by improved hexamerization.

We also studied the effect of galactosylation on IgG4 complement activation, which has previously received little attention, as most of the research on Fc glycosylation has focused on IgG1. For IgG1 it has been shown that galactosylation enhances C1q binding and downstream CDC activity (20, 21, 23, 52). Compared to IgG1, the IgG4 glycovariants in our study only showed significant differences in overall activation at the level of C3b deposition, with the high galactose variants performing better than the low galactose or high mannose variants, but the mode of activation was still similar for all variants studied. Possibly, galactosylation of IgG4 may improve its ability to hexamerize as has been shown for IgG1 (20, 21, 23, 52) and can therefore potentiate complement activation by IgG4 *via* the classical pathway. A slight impairment of complement activation by IgG4 was found in the presence of Fab glycans. IgG Fab glycosylation impacts antibody stability, half-life, binding characteristics, and immune complex formation (69). It has been demonstrated that IgG4 Fab glycosylation is significantly increased compared to total IgG (24). Since IgG4 antibodies are poorer activators of effector functions than other IgG subclasses, elevated levels of Fab glycan might impact the binding of IgG4 to effector molecules, such as C1q, and potentially the formation of hexamers.

Another important hallmark of IgG4 is its ability to undergo Fab arm exchange and form bispecific antibodies (14–16), which has not been considered in any of the previous studies which used mainly recombinant monoclonal antibodies (4, 21, 59–61). The Fab arm exchange of IgG4 may have a physiological role as natural bi-specific molecules cannot cross-link antigen or elicit lymphoid responses, may dampen inflammatory responses (16, 70) and potentially reduce complement activation. Most of the normal human plasma IgG4 molecules are bispecific (71, 72). It has been shown that within the total serum of healthy inndividuals, the hybrid IgG4λ/κ antibodies fraction accounts for up to 33%. Most likely the remaining IgG4 with the same light chain on both Fab arms may also have undergone half-molecule exchange at some point (72). Bispecific IgG4 molecules are widespread in normal serum and possibly represents the typical configuration and even therapeutical IgG4 molecules can undergo Fab arm exchange with serum IgG4 (73). Therefore, studies relying on monovalent antibodies may not be reflective of the *in vivo* situation. Here we show that bispecific IgG4 induces less C3b deposition than monospecific IgG4, which is particularly relevant for interpreting other studies on IgG4 complement activation that use monospecific antibody preparations. Interestingly, it has been previously shown that a monovalent IgG1 antibody outperformed its bivalent monospecific counterpart in CDC experiments (18, 74). However, for another IgG1 antibody tested, both the monospecific and bispecific antibodies performed similarly (18), suggesting the effect may also be dependent on specific antigen-antibody interactions, like we have seen in our studies. This indicates that the Fab arm exchange of IgG4 molecules may contribute to the limited IgG4-mediated complement activation *in vivo*.

IgG4 is believed to have mainly neutralizing and anti-inflammatory functions due to limited complement fixation and crosslinking, therefore it has been exploited for the development of therapeutic antibodies that are intended to block their target, often including a stabilizing S228P mutation to minimize *in vivo* Fab arm exchange. Currently IgG4 antibodies account for approximately 13% of all therapeutic monoclonal antibodies approved or in development (75). Two examples are the humanized kappa IgG4 antibodies pembrolizumab and nivolumab, which are both programmed death receptor-1 (PD1)-blocking antibodies (76, 77). However, our results show that the ability of monospecific IgG4 to activate complement should be taken into consideration when developing new therapeutics binding membrane-bound targets that are administered at high concentrations. As a blocking antibody, a monovalent (bispecific) monoclonal antibody might be considered if unwanted complement activation might be an issue.

In summary, we demonstrate that although IgG4 is a weak trigger for complement activation, it cannot be considered completely ‘complement-dead’, since it does activate complement, but only at high antigen densities and high antibody concentrations, and only *via* the classical pathway. In addition, galactosylation has a subtle enhancing impact on the capacity of IgG4 antibodies to activate complement, whereas Fab arm exchange, a hallmark of IgG4, has the opposite effect. These findings imply that pathogenic role of IgG4 in diseases such as IgG4-RD or pMN *via* recruitment of the complement system is possible, but unlikely.

## Data availability statement

The raw data supporting the conclusions of this article will be made available by the authors, without undue reservation.

## Author contributions

All authors contributed to the article and approved the submitted version. Designing research studies: NO, TD, TR, GV. Conducting experiments and data acquisition: NO, TD, MS, PO-H, JN, CK, MW. Analyzing data: NO, TD, MS, JC, TR. Writing the manuscript: NO, TD, TR.
