# Supplementary material for: Factors affecting IgG4-mediated complement activation
Source: Front Immunol. 2023 Jan 26;14:1087532. doi: 10.3389/fimmu.2023.1087532 (PMC9910309; doi:10.3389/fimmu.2023.1087532)
Supplement: Supplementary file 1 [file DataSheet_1.pdf]

Table S1. IgG glycopeptides included in the final analyte list. H: hexose, N: N-acetylhexosamine, F: fucose, S: N-acetylneuraminic acid (sialic acid). The theoretical *m/z* values of monoisotopic peaks are given.

**Glycan composition**	**IgG Subclass**	**[M+3H]$^{3+}$**
**IgGIVH5N2**	IgG4	797,3182
**IgGIVH3N3F1**	IgG4	805,6621
**IgGIVH3N4**	IgG4	824,6692
**IgGIVH4N3F1**	IgG4	859,6797
**IgGIVH5N3**	IgG4	865,0113
**IgGIVH3N4F1**	IgG4	873,3552
**IgGIVH5N3F1**	IgG4	913,6973
**IgGIVH6N3**	IgG4	919,0289
**IgGIVH4N4F1**	IgG4	927,3728
**IgGIVH5N4**	IgG4	932,7044
**IgGIVH3N5F1**	IgG4	941,0483
**IgGIVH4N3F1S1**	IgG4	956,7115
**IgGIVH5N3F2**	IgG4	962,3832
**IgGIVH6N3F1**	IgG4	967,7149
**IgGIVH4N4F2**	IgG4	976,0588
**IgGIVH5N4F1**	IgG4	981,3904
**IgGIVH6N4**	IgG4	986,7220
**IgGIVH4N5F1**	IgG4	995,0659
**IgGIVH3N6F1**	IgG4	1008,7414
**IgGIVH5N3F1S1**	IgG4	1010,7291
**IgGIVH6N3S1**	IgG4	1016,0607
**IgGIVH4N4F1S1**	IgG4	1024,4046
**IgGIVH5N4F2**	IgG4	1030,0764
**IgGIVH6N4F1**	IgG4	1035,4080
**IgGIVH5N5F1**	IgG4	1049,0835
**IgGIVH6N3F1S1**	IgG4	1064,7467
**IgGIVH5N4F1S1**	IgG4	1078,4222
**IgGIVH6N4S1**	IgG4	1083,7538
**IgGIVH4N5F1S1**	IgG4	1092,0977
**IgGIVH6N5F1**	IgG4	1103,1011
**IgGIVH5N4F2S1**	IgG4	1127,1082
**IgGIVH5N4F1S2**	IgG4	1175,4540

Table S2. IgG glycopeptides included in the final analyte list for the anti-biotin antibodies

**Glycan composition**	**IgG Subclass**	**[M+1H]1+**	**[M+2H]2+**	**[M+3H]3+**	**[M+4H]4+**
IgGI1H3N3	IgG1	2284,9086	1142,9579	762,3077	571,9826
IgGI1H3N4	IgG1	2487,9880	1244,4976	830,0008	622,7524
IgGI1H4N3	IgG1	2446,9614	1223,9843	816,3253	612,4958
IgGI1H4N4	IgG1	2650,0408	1325,5240	884,0184	663,2657
IgGI1H5N4	IgG1	2812,0936	1406,5504	938,0361	703,7789
IgGI1H3N3F1	IgG1	2430,9665	1215,9869	810,9937	608,4971
IgGI1H3N4F1	IgG1	2634,0459	1317,5266	878,6868	659,2669
IgGI1H4N3F1	IgG1	2593,0193	1297,0133	865,0113	649,0103
IgGI1H4N4F1	IgG1	2796,0987	1398,5530	932,7044	699,7801
IgGI1H5N3F1	IgG1	2755,0722	1378,0397	919,0289	689,5235
IgGI1H5N4F1	IgG1	2958,1515	1479,5794	986,7220	740,2933
IgGI1H3N5F1	IgG1	2837,1253	1419,0663	946,3799	710,0368
IgGI1H4N5F1	IgG1	2999,1781	1500,0927	1000,3975	750,5500
IgGI1H3N3F1S1	IgG1	2722,0619	1361,5346	908,0255	681,2709
IgGI1H4N3F1S1	IgG1	2884,1147	1442,5610	962,0431	721,7841
IgGI1H5N3F1S1	IgG1	3046,1676	1523,5874	1016,0607	762,2973
IgGI1H4N4F1S1	IgG1	3087,1941	1544,1007	1029,7362	772,5540
IgGI1H5N4F1S1	IgG1	3249,2469	1625,1271	1083,7538	813,0672
IgGI1H5N4F1S2	IgG1	3540,3424	1770,6748	1180,7856	885,8410
IgGI1H5N5F1S2	IgG1	3743,4217	1872,2145	1248,4788	936,6109
IgGI1H5N2	IgG1	2405,9349	1203,4711	802,6498	602,2392
IgGI1H6N2	IgG1	2567,9877	1284,4975	856,6674	642,7524
IgGI1H7N2	IgG1	2730,0405	1365,5239	910,6850	683,2656
IgGIV1H3N3	IgG4	2268,9137	1134,9605	756,9761	567,9839
IgGIV1H3N4	IgG4	2471,9931	1236,5002	824,6692	618,7537
IgGIV1H4N3	IgG4	2430,9665	1215,9869	810,9937	608,4971
IgGIV1H4N4	IgG4	2634,0459	1317,5266	878,6868	659,2669
IgGIV1H5N4	IgG4	2796,0987	1398,5530	932,7044	699,7801
IgGIV1H3N3F1	IgG4	2414,9716	1207,9894	805,6621	604,4984
IgGIV1H3N4F1	IgG4	2618,0510	1309,5291	873,3552	655,2682
IgGIV1H4N3F1	IgG4	2577,0244	1289,0159	859,6797	645,0116
IgGIV1H4N4F1	IgG4	2780,1038	1390,5555	927,3728	695,7814
IgGIV1H5N3F1	IgG4	2739,0773	1370,0423	913,6973	685,5248
IgGIV1H5N4F1	IgG4	2942,1566	1471,5820	981,3904	736,2946
IgGIV1H3N5F1	IgG4	2821,1304	1411,0688	941,0483	706,0380
IgGIV1H4N5F1	IgG4	2983,1832	1492,0952	995,0659	746,5513
IgGIV1H3N3F1S1	IgG4	2706,0670	1353,5371	902,6939	677,2722
IgGIV1H4N3F1S1	IgG4	2868,1198	1434,5636	956,7115	717,7854
IgGIV1H5N3F1S1	IgG4	3030,1727	1515,5900	1010,7291	758,2986
IgGIV1H4N4F1S1	IgG4	3071,1992	1536,1032	1024,4046	768,5553
IgGIV1H5N4F1S1	IgG4	3233,2520	1617,1297	1078,4222	809,0685
IgGIV1H5N4F1S2	IgG4	3524,3475	1762,6774	1175,4540	881,8423
IgGIV1H5N5F1S2	IgG4	3727,4268	1864,2171	1243,1471	932,6122
IgGIV1H5N2	IgG4	2389,9400	1195,4736	797,3182	598,2404
IgGIV1H6N2	IgG4	2551,9928	1276,5000	851,3358	638,7537
IgGIV1H7N2	IgG4	2714,0456	1357,5264	905,3534	679,2669

Table S3. Description and calculation of IgG glycosylation traits. H: hexose, N: N-acetylhexosamine, F: fucose, S: N-acetylneuraminic (sialic) acid

	**Description**	**Formula**
**IgG1 bisection**	*N*-glycans carrying a bisected *N*-acetylglucoseamine	(H5N5F1S1 + H4N5F1 + H5N5F1) / sum of all IgG1 glycopeptides
**IgG1 galactosylation**	*N*-glycans carrying galactose(s)	(1/2 * (H4N4 + H4N4F1 + H4N5F1 + H4N4F1S1) + 2/2 * (H5N4 + H5N4F1 + H5N4S1 + H5N5F1S1 + H5N4F1S2 + H5N5F1 + H5N4F1S1)) / sum of all glycopeptides
**IgG1 sialylation**	*N*-glycans carrying *N*-acetylneuraminic (sialic) acid(s)	(1/2 * (H5N4S1 + H5N5F1S1 + H4N4F1S1) + 2/2 * H5N4F1S2) / sum of all IgG1 glycopeptides
**IgG1 fucosylation**	*N*-glycans carrying a core fucose	(H3N4F1 + H4N4F1 + H5N4F1 + H5N5F1S1 + H5N4F1S2 + H4N5F1 + H4N4F1S1 + H5N5F1S1 + H5N4F1S1) / sum of all IgG1 glycopeptides

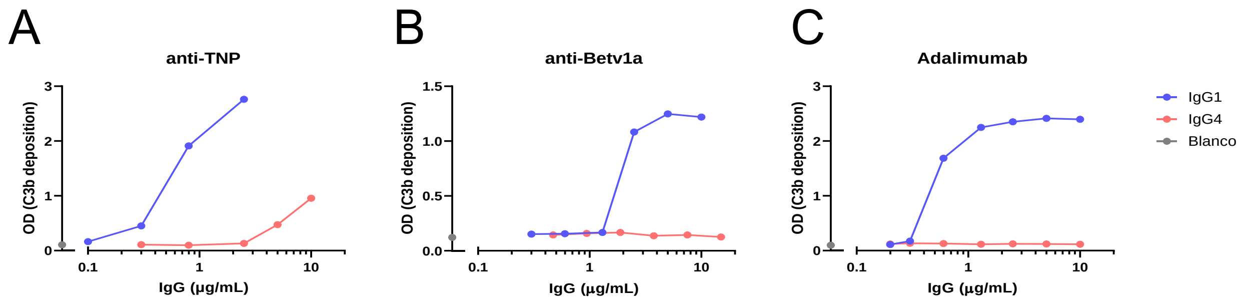

Supplement figure 1. No IgG4-mediated complement activation observed in presence of protein antigens. C3b deposition by **(A)** anti- anti-trinitrophenyl (TNP), **(B)** anti-Betv1a and **(C)** adalimumab IgG1 and IgG4 in the presence of human serum albumin (HSA)-TNP (1 mM; 10 µg/mL), Betv1a (5 µg/mL) or anti-adalimumab Fabs (5 µg/mL) and 2.5% human serum.

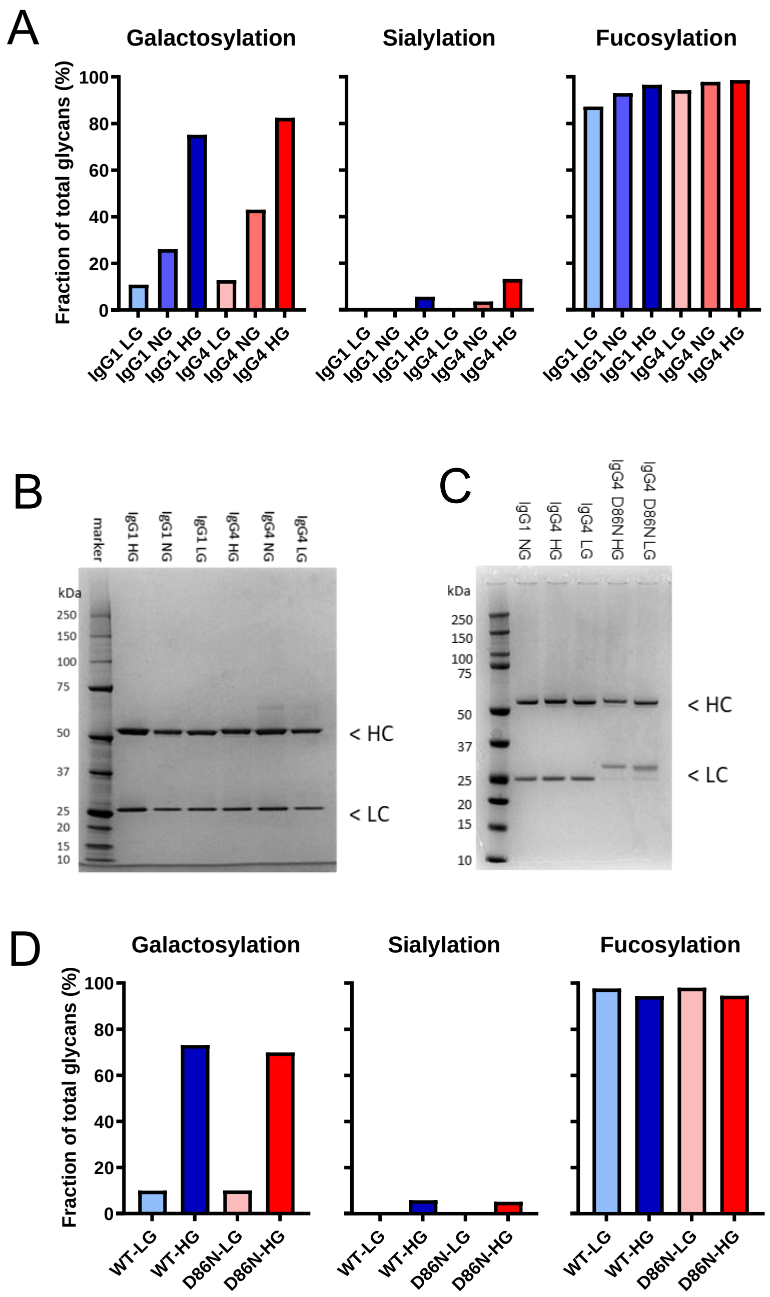

Supplement figure 2. IgG1 and IgG4 glycovariants show similar glycosylation profiles. (A) Glycosylation patterns of glyco-engineered IgG1 and IgG4 Fc with high (HG), normal (NG) and low (LG) galactose composition. **(B)** IgG1 and IgG4 galactose-glycovariants and **(C)** IgG4 Fab variants were protein size separated via SDS-PAGE under reducing conditions. The molecular weight marker includes bands from 10 to 250 kDa. HC, heavy chain; LC, light chain. **(D)** Glycosylation patterns of IgG4 Fab variants, wild type IgG4, and a variant with D86N substitution in the light chain to introduce an additional glycosylation site, were profiled. Shown are fractions of galactose, sialic acid, and core fucose of total glycans.

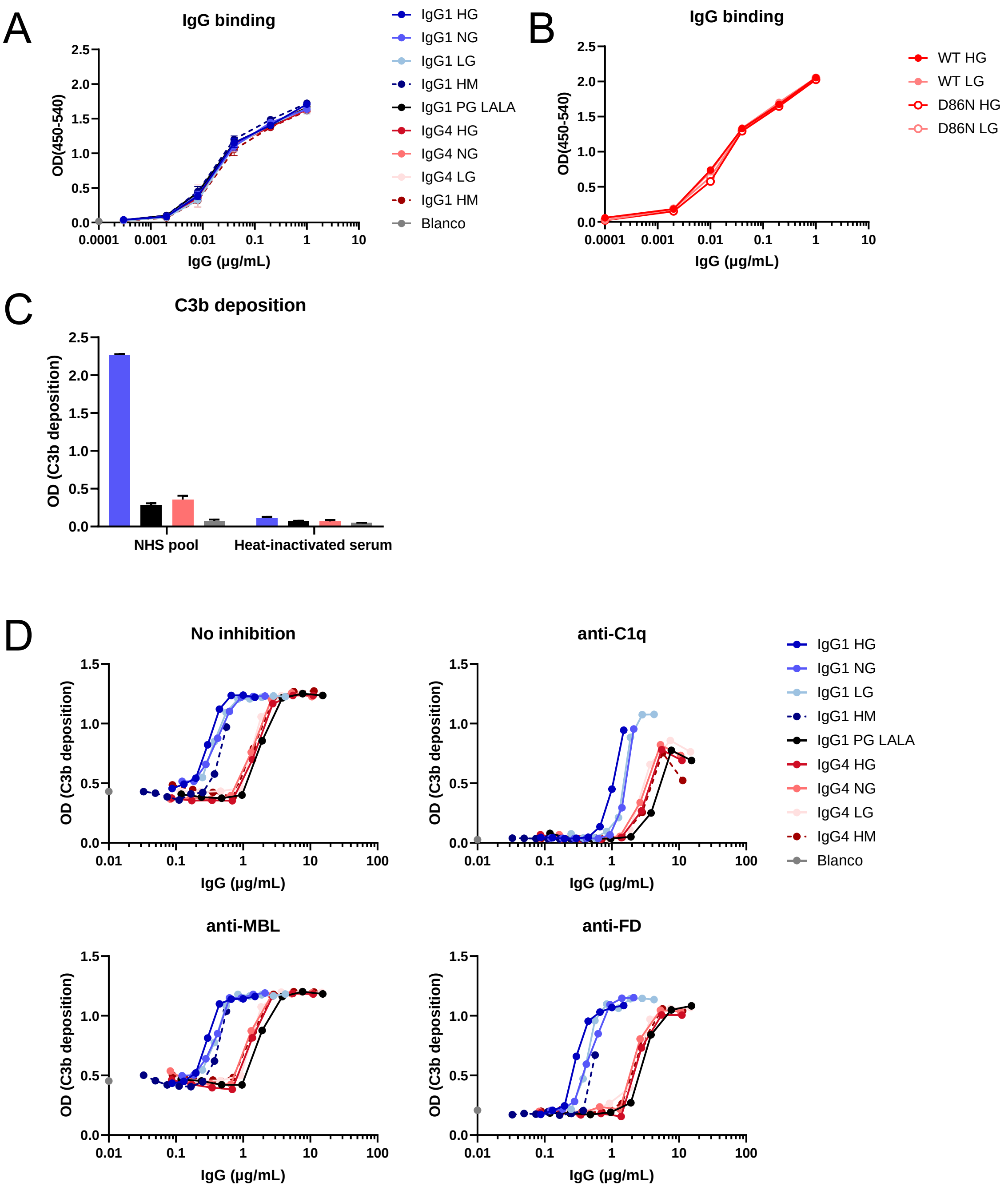

Supplement figure 3. Controls for complement assays. **(A)** IgG binding of all glycovariants and **(B)** Fab glycovariants was tested to identify concentration variations. **(C)** To test unspecific C3b deposition, anti-biotin IgG1, IgG4 and IgG1 PG-LALA (eliminated Fc-mediated effector functions) were tested in the presence of biotinylated human serum albumin (HSA; 240 µM biotin) and 2.5% normal or heat-inactivated human serum. **(D)** C3b deposition by the glycovariants was also measured at 10% NHS and 37°C incubation.

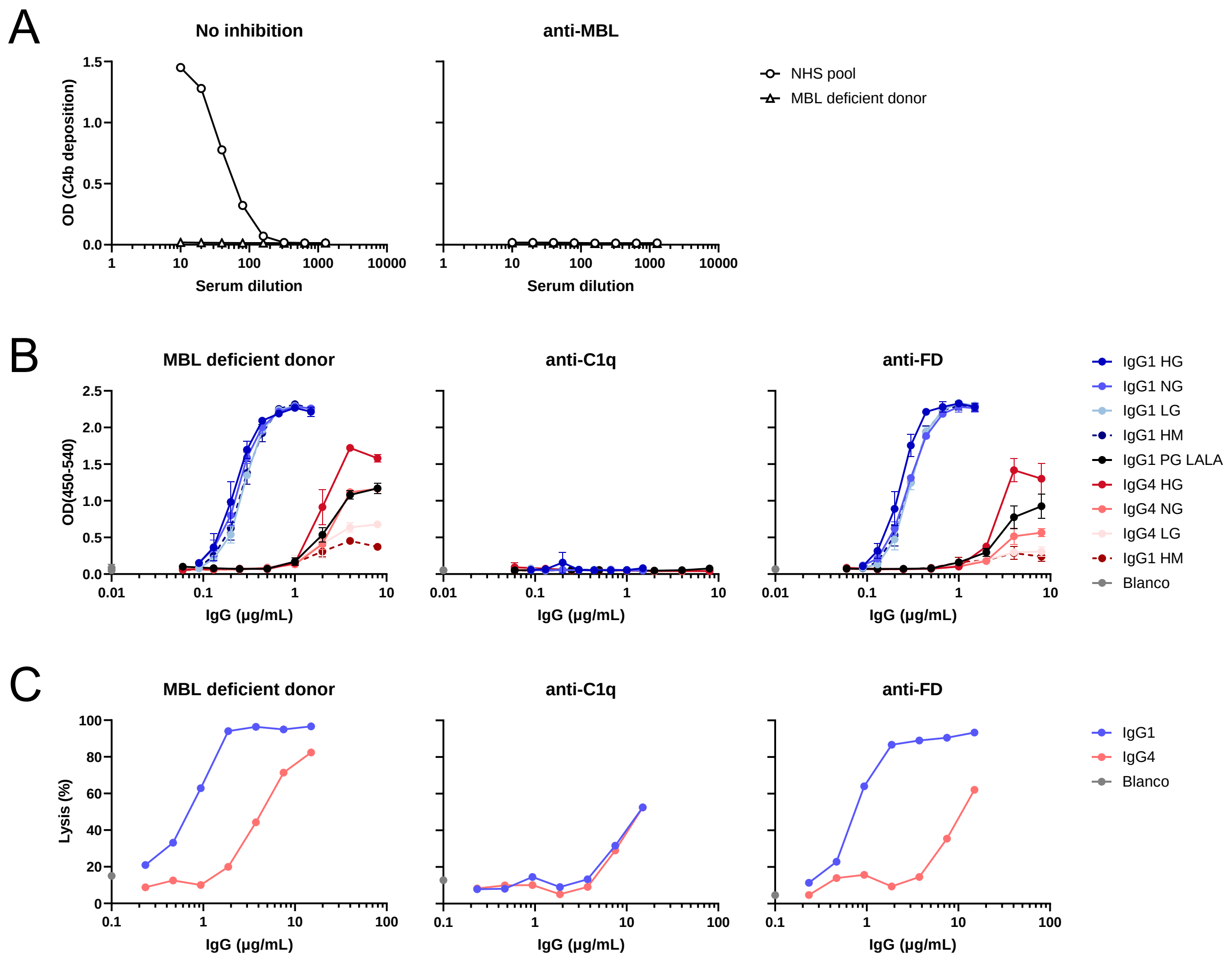

Supplement figure 4. Inhibition of mannose-binding lectin (MBL) does not affect antibody-mediated complement activation. (A) The activity of the lectin pathway was tested for a normal healthy serum pool and serum from a MBL deficient individual. Serial dilutions of both sera were incubated on mannan coated plates (50 µg/mL) in the absence or presence of MBL inhibitor, after which deposited C4b was detected. **(B)** C3b deposition induced by anti-biotin IgG1 and IgG4 glycovariants (high mannose (HM), high galactose (HG), normal galactose (NG) and low galactose (LG)), and IgG1 PG-LALA in the presence of in presence or absence of either C1q or FD inhibitors biotinylated human serum albumin (240 µM biotin) and 2.5% MBL-deficient serum. **(C)** Complement-mediated lysis of biotinylated human red blood cells (2.5 mM biotin) by IgG1 and IgG4 in presence or absence of either C1q, MBL, or FD inhibitors (full monoclonal antibodies and Fab fragments) and 10% MBL-deficient serum.
